# Beneficial Effects of the Novel Digital Incentive Spirometer Device and Incentive Spirometer in Patients Undergoing Open-Heart Surgery: Randomized Controlled Trial

**DOI:** 10.2196/68158

**Published:** 2025-05-06

**Authors:** Kornanong Yuenyongchaiwat, Somrudee Harnmanop, Lucksanaporn Mahawong, Nattapoomin Ruangphet, Kannika Jeangubon, Chaopraya Nenmanee, Chitima Kulchanarat, Opas Satdhabudha

**Affiliations:** 1 Physiotherapy Department, Faculty of Allied Health Sciences, Thammasat University, 99 Moo 18, Paholyothin Road, Klong Luang, Rangsit, Pathumtani, 12121, Thailand, 66 84521680; 2 Thammasat University Research Unit for Physical Therapy in Respiratory and Cardiovascular Systems, Thammasat University, Pathumtani, Thailand; 3 Physical Therapy Center, Thammasat University Hospital, Pathumtani, Thailand; 4 Department of Surgery, Faculty of Medicine, Thammasat University, Pathumtani, Thailand

**Keywords:** digital incentive spirometer device, flow-oriented incentive spirometer, open-heart surgery, randomized controlled trial, respiratory function

## Abstract

**Background:**

The number of patients undergoing open-heart surgery (OHS) is persistently increasing. Additionally, postoperative pulmonary complications have been reported after OHS, and an incentive spirometer has been suggested to prevent postoperative pulmonary complications. However, no commercial incentive spirometer provides the precise inhalation volume. We developed a digital incentive spirometer (DIS) that displays the relevant data.

**Objective:**

In this study, we aimed to explore the beneficial effects of a DIS on respiratory function in patients who underwent OHS.

**Methods:**

A randomized controlled trial was designed with 32 patients scheduled for OHS: 16 individuals each were assigned to the DIS and the flow-oriented incentive spirometer (ie, Triflow incentive spirometer) groups, respectively. The patients were requested to use the DIS and Triflow incentive spirometer 15 times/set, two sets/day, from day 1 to 5 postextubation. All participants underwent lung function and respiratory muscle strength assessments prior to OHS and on day 5 postextubation postoperatively. For comparison between and within the groups, we performed an intention-to-treat analysis with a two-way mixed analysis of variance.

**Results:**

In both the DIS and Triflow incentive spirometer groups, pulmonary function parameters and maximal respiratory pressure were markedly reduced on day 5 postextubation when compared with those prior to OHS (*P*<.05). There were no significant differences in pulmonary function or respiratory muscle strength between the two groups (*P*>.05).

**Conclusions:**

Pulmonary function and respiratory muscle strength did not differ significantly between the DIS and Triflow incentive spirometer groups among patients who underwent OHS.

## Introduction

An incentive spirometer is typically used for patients with pulmonary issues and those at risk of postoperative pulmonary complications. This device exercises the lungs and necessitates deep breathing, requiring patients to inhale slowly and deeply to increase the volume of air entering the lungs [1,2].

A health professionals’ survey on the perspective on the utility of an incentive spirometer among nursing and respiratory care staff reported that healthcare professionals agreed that an incentive spirometer is essential for patient care (92.7%) [3]. Furthermore, healthcare professionals believed that an incentive spirometer could improve pulmonary function (92.0%) and inspiratory capacity (93.0%). An incentive spirometer can also help prevent and reverse atelectasis (96.6% and 90.0%, respectively) and prevent and reverse pneumonia (92.5% and 68.4%, respectively) [3]. Among patients who underwent lung surgery, the incidence of postoperative pulmonary complications was lower in those who used an incentive spirometer preoperatively and postoperatively than in those who did not use an incentive spirometer [4]. Thus, the use of an incentive spirometer pre- and postheart surgery can reduce the risk of postoperative pulmonary complications or hospitalization [1,5,6].

A review of the literature published between 2010 and 2021 revealed that the average total cardiac surgical volume in high-income countries was 123.2 per 100,000 people annually, which included coronary artery bypass graft (CABG) and valvular heart disease (36.7 and 30.8 per 100,000 people per year, respectively). In high-middle-income countries including Thailand, the average total cardiac surgical volume was 86.1 per 100,000 people annually, with CABG and valvular heart disease reaching values of 35.4 and 13.3/per 100,000 people per year [7].

Among patients undergoing CABG, the use of incentive spirometer devices can reduce the duration of postoperative mechanical ventilation and shorten hospitalization [6]. Additionally, both flow- and volume-oriented incentive spirometer devices can improve pulmonary function (ie, the forced vital capacity [FVC], forced expiratory volume in the 1st second [FEV1], and the peak expiratory flow rate [PEFR]) in patients undergoing surgery [8].

Both volume- and flow-oriented incentive spirometer devices reflect respiratory performance and capacity and encourage improvements in deep inspiration. However, these devices fail to provide a specific volume or airflow of inspiration; for example, the flow-oriented incentive spirometer is designed to improve lung volumes and employs a flow-controlled mechanism using three floating balls. The device displays airflow at 600, 900, and 1200 ml/s as three different colored balls. Additionally, some patients may be unable to elevate their floating balls, which may lead to a lack of motivation.

Therefore, we developed a digital incentive spirometer (DIS) capable of displaying both the volume of airflow entering the lungs and the volume of air inhaled as numerical indicators, graphical representations, colors, and auditory signals. Furthermore, we explored the benefit of DIS on the respiratory performance of patients who underwent open-heart surgery (OHS).

## Methods

### Study Design

The study was twofold. The first part aimed to design and develop the DIS. The second part was designed as a randomized control trial to explore the benefits of DIS on pulmonary function and respiratory muscle strength.

### Ethical Considerations

The protocol of the study was approved by the Ethics Human Committee of Thammasat University based on the Declaration of Helsinki, the Belmont report, CIOMS guidelines and the International practice (ICH-GCP) with COA No. 074/2566. The study followed the 2010 CONSORT Reporting guidelines (Checklist 1). Informed consent was obtained from all subjects involved in the study. Written informed consent has been obtained from the participants to publish this paper.

### Design and Development

We designed and developed the DIS based on the principles of differential pressure flow meters. The MPX10DP (NXP Semiconductors, Thailand Co., Ltd) is a pressure sensor designed to measure pressures ranging between 0 and 10 kPa or 0 to 1.45 psi. It operates by comparing differential inputs to determine the pressure and provides an analog output signal. The circuit design utilizes the ESP32 microcontroller to read the analog signals from the MPX10DP sensor. In addition, an LM358N operational amplifier was employed to amplify the output signal and convert the analog signal into electrical voltage. Therefore, this voltage was used to calculate the pressure, which was then compared with measurements obtained from the AWM720P1 airflow sensor. Furthermore, the AWM720P1 airflow sensor is a standard device to measure airflow, expressed in units of standard liters per minute. The AWM720P1 airflow sensor has low hysteresis and repeatability errors (less than 0.35% of reading), providing better system accuracy. This sensor employs a microcontroller to read the sensor data, convert the analog signal into an electrical voltage, and compute the airflow rate, which is the output in L/s. The conversion of pressure to airflow is achieved by correlating the pressure readings obtained from the MPX10DP sensor with the airflow measurements from the AWM720P1 sensor. This is accomplished by integrating both sensors into a unified system and recording data concurrently to determine the linear relationship between pressure and airflow.

According to the concept of air passing through different cross-sectional areas and air pressures, the airflow from the prototype DIS device into the lungs passes through a tube with varying cross-sectional areas and air pressures. Therefore, the air volume was transmitted to the sensor and calculated using a microcontroller that has an analog-to-digital conversion module.

**Figure 1.**
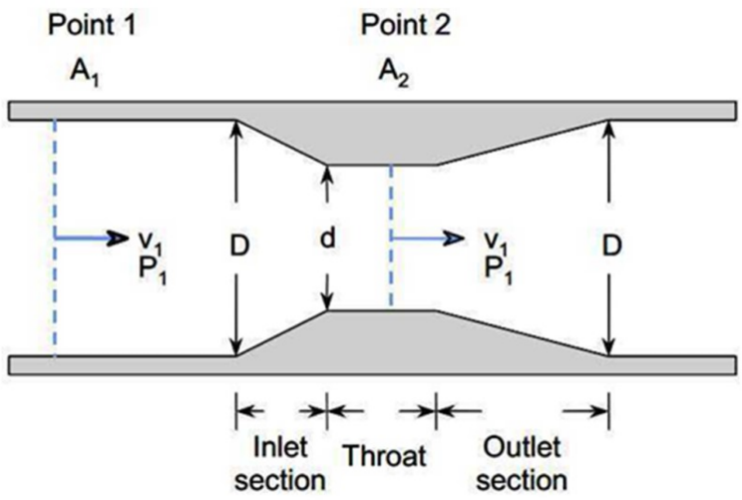
Principles of the continuity equation and the Bernoulli equation.

The principle of flow rate measurement involves generating a pressure drop within the tube through which the fluid flows using the principles of the continuity equation and the Bernoulli equation. The continuity equation can be summarized as the product of the cross-sectional area of the tube and the flow velocity of an ideal fluid, which remains constant at any position within the tube. This relationship ensures that the airflow is consistent according to the flow equation (Figure 1),


$$Q=v_{1}\times A_{1}=v_{2}\times A_{2}$$

where v denotes the mean velocity, A denotes the cross-section area normal to the direction of flow, and Q denotes the discharge or volume flow rate of water flowing at a given rate of time through any section of the area.

**Figure 2.**
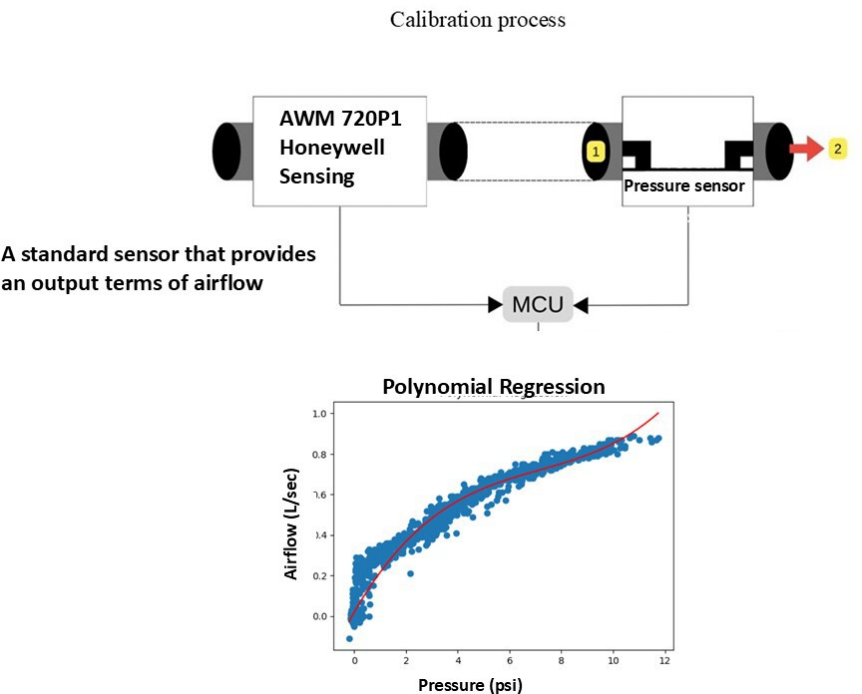
Calibration of the digital incentive spirometer. MCU: microcontroller.

The DIS was calibrated via the sensor, and the air pathways were connected in series between the prototype DIS and the industrial sensor. The readings acquired from the DIS prototype were in the form of pressure levels (analogs), which were compared with the known airflow rates from the industrial-grade sensor, referenced from the characteristic graph shown in Figure 2. Air was drawn at various levels during data collection to obtain sufficient data to create a regression equation model, which was used to determine the airflow rate from the electrical potential input obtained from the differential pressure sensor, as follows:

$0.01967741+(0.22080302\times {\text{pressure}}_{1})+(-0.0257367\times {\text{pressure}}_{2})+(0.00119711\times {\text{pressure}}_{3})$

Therefore, a DIS device consists of the following three main components: (1) incentive spirometer device: This device includes a mouthpiece, tubing, and a main unit, and captures the inhaled air of the user. (2) Display screen: This includes a color bar, airflow volume levels, and a display of the air volume, and provides visual feedback to the volunteers undergoing the test. (3) Pressure sensor: The pressure sensor MPX10DP measures the inhalation pressure via the mouth, and it is highly accurate and reliable for a differential pressure flow meter type.

**Figure 3.**
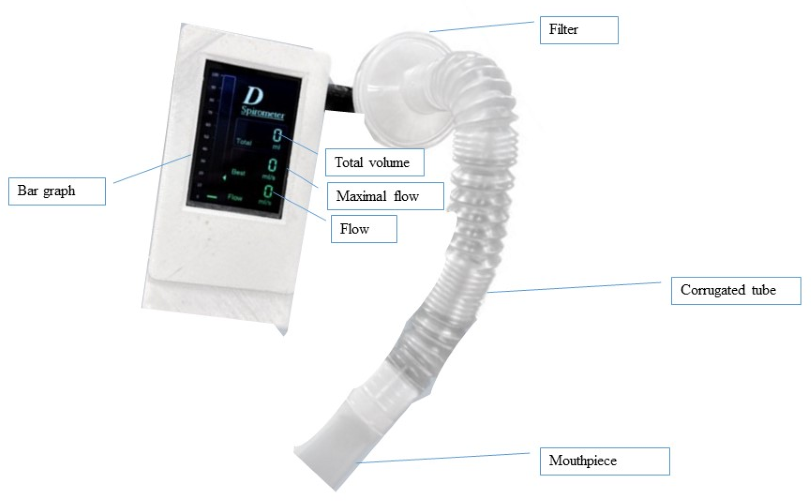
Digital incentive spirometer device.

Volunteers can read the airflow volume readings while performing breathing exercises using the prototype DIS. The air volume display screen presents the volume of air inhaled and exhaled during each breathing cycle (Figure 3).

### Experimental Design

We explored the benefits of using the DIS. In this study, we recruited patients who underwent OHS at Thammasat University Hospital. Patients who were scheduled to undergo CABG or valvular surgery, aged 35‐80 years, both men and women, were enrolled. Patients with a history of heart surgery or arrhythmia 24 hours prior to the test and those with active respiratory infection (eg, tuberculosis, COVID-19, or multiple drug resistance) were excluded. In addition, the discontinuation criteria were patients who required reintubation, those with postsurgical infection, those with prolonged intubation exceeding 72 hours, and those who needed admission to the hospital less than 5 days after extubation.

The sample size was calculated by using G-power 3.1.9.4, the effect size was set at 0.25, the statistical power was 0.80, and the alpha was .05; therefore, a total of 28 participants were examined initially. However, to avoid missing data or to adjust for potential dropouts, 32 randomly selected participants were finally enrolled. All participants were provided with an information sheet, and a consent form was signed before participation.

All participants were required to undergo pulmonary function evaluation (ie, the predicted %FVC and the predicted PEFR) by using CareFusion MicroLab (a Micro Direct company, United Kingdom) and respiratory muscle strength tests (maximal inspiratory pressure [MIP] and maximal expiratory pressure [MEP]) measured by a MicroRPM Respiratory Pressure Meter (Micro Medical Micro RPM., United Kingdom) prior to OHS and on day 5 postextubation postoperatively. A standard protocol for pulmonary function and respiratory muscle strength testing was followed according to the recommendations of the European Respiratory Society (ERS)/American Thoracic Society technical standard and ERS statement on respiratory muscle testing, respectively [9-11]. A single-blind randomized clinical trial was conducted with two assessors who were physical therapists.

**Figure 4.**
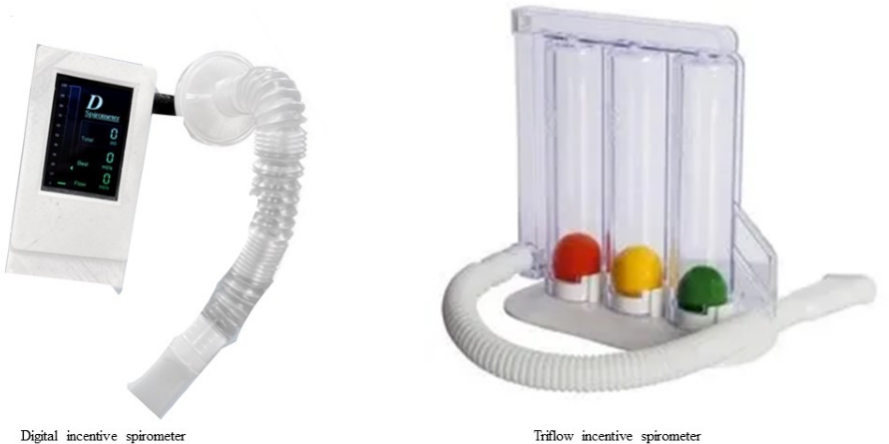
Digital incentive spirometer and Triflow incentive spirometer.

The selected 32 participants were assigned to use the DIS or Triflow incentive spirometer (control group; Figure 4). The patients were required to use the DIS and Triflow incentive spirometer 15 times/set and perform two sets daily (ie, in the morning and evening), from day 1 to 5 postextubation. All participants were instructed on how to use the DIS or Triflow incentive spirometer via an in-person demonstration by a physical therapist who was not the assessor. The participant was required to sit on the bed or upright position and hold the device (ie, DIS or Triflow incentive spirometer). After that, they were asked to breathe out normally and then take a deep breath in via the mouthpiece and try to hold the breath for 3‐5 seconds or as long as possible. During the intervention, all participants were supervised by two physical therapists, while other physical therapists were assigned to be assessors. In addition, those participants underwent physical therapy protocol, for example, breathing exercises, cough training with pillow support, calisthenics exercise, and ambulation (sitting, standing, and walking). The participants received physical therapy once a day until discharged from the hospital.

### Statistical Analysis

Data were analyzed using IBM SPSS Statistics version 24.0. The Shapiro-Wilk test was used for assessing normal data distribution. An intention-to-treat analysis with a two-way mixed analysis of variance (time (2) × type (2)) was used to compare between and within groups for pulmonary function and respiratory muscle strength. Statistical significance was set at *P*<.05.

## Results

### Patient Classification

**Figure 5.**
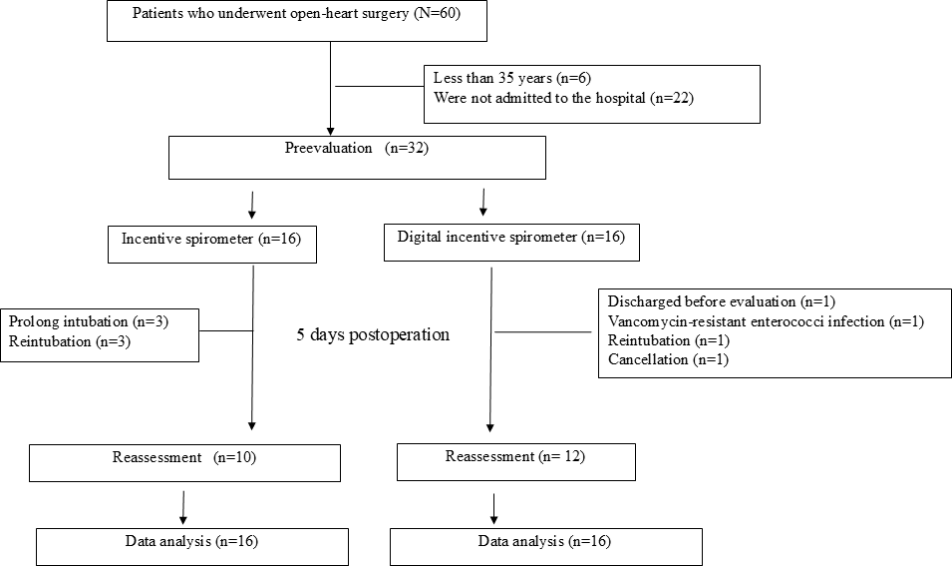
Recruitment of participants.

Sixty patients scheduled for OHS were included in the initial study. Unfortunately, 6 patients were excluded because they were <35 years of age, and 22 participants were included in the study during the initial session; however, due to the cancellation of their surgery, they were excluded from the study. Ultimately, 32 patients were enrolled in the study. A simple random sampling (ie, using a random lottery) was performed, and the patients were divided into two groups based on the device used: the DIS group (n=16) and Triflow incentive spirometer group (n=16). All participants underwent pulmonary function tests (ie, the predicted %FVC and the predicted %PEFR) and respiratory muscle strength (MIP and MEP) assessment prior to surgery. Twenty-two patients were reassessed on day 5 postextubation. Of the 10 patients who did not complete the test, 3 required prolonged intubation (ie, greater than 72 hours), 4 underwent reintubation, and 1 was discharged before the assessment; surgery was canceled in the final excluded patient (n=1; Figure 5).

Of the 36 patients who underwent OHS, 11 male and 21 female patients were included in the current study (Table 1). Considering patients assigned to the DIS and Triflow incentive spirometer groups, there were no significant differences in terms of age, comorbidities, type of operation, pulmonary function, and respiratory muscle strength (Table 2).

**Table 1.** Characteristics of patients who underwent open-heart surgery by group.

	Total (n=32), n (%)	DIS^a^ (n=16), n (%)	Triflow IS^b^ (n=16), n (%)	Chi-square *(df)*	*P* value
Sex, n (%)				0.139 (1)	.710
Male	11 (34.38)	5 (45.45)	6 (54.55)		
Female	21 (65.63)	11 (52.38)	10 (47.62)		
Diabetes mellitus, n (%)				3.137 (1)	.077
Yes	15 (46.88)	10 (66.67)	5 (33.33)		
No	17 (53.13)	6 (35.29)	11 (64.71)		
Hypertension, n (%)				0.183 (1)	.669
Yes	7 (21.88)	4 (57.14)	3 (42.86)		
No	25 (78.13)	12 (48.00)	13 (52.00)		
Dyslipidemia, n (%)				0.000 (0)	>.99
Yes	14 (43.75)	7 (50.00)	7 (50.00)		
No	18 (56.25)	9 (50.00)	9 (50.00)		
Surgery, n (%)				1.077 (1)	.584
CABG^c^	18 (56.25)	9 (50.00)	9 (50.00)		
Valvular surgery	13 (40.63)	6 (46.15)	7 (53.85)		
Combined	1 (3.13)	1 (100.00)	0 (0.00)		

a DIS: digital incentive spirometer.

b IS: incentive spirometer.

c CABG: coronary artery bypass graft.

**Table 2.** Patient characteristics considering respiratory function.

Characteristic	Total (n=32)	DIS^a^ (n=16)	Triflow IS^b^ (n=16)	*t* test *(df)*	P value
Age (y), mean (SD)	62.25 (10.98)	63.75 (11.93)	66.75 (10.10)	−0.768 (30)	0.449
MIP^c^ (cmH2O), mean (SD)	44.47 (26.99)	42.69 (31.06)	46.25 (23.11)	−0.368 (30)	0.715
MEP^d^ (cmH2O), mean (SD)	38.81 (20.44)	33.69 (17.43)	43.94 (22.45)	−1.443 (30)	0.159
FVC^e^ %, mean (SD)	69.63 (21.42)	68.25 (19.73)	62.50 (16.73)	0.889 (30)	0.381
PEFR^f^ %, mean (SD)	46.09 (20.92)	47.56 (22.05)	44.63 (20.34)	0.392 (30)	0.698

a DIS: digital incentive spirometer.

b IS: incentive spirometer.

c MIP: maximal inspiratory pressure.

d MEP: maximal expiratory pressure.

e FVC: forced vital capacity.

f PEFR: peak expiratory flow rate.

### Effect of the Intervention on Pulmonary Function and Respiratory Muscle Strength

Both groups exhibited markedly reduced pulmonary function and respiratory muscle strength on day 5 postextubation when compared with preoperative values (*P*<.05). However, patients who underwent DIS showed a slower decline in respiratory performance when compared with those who used the Triflow incentive spirometer device for MIP (mean difference [SE], −8.13 [3.86], *P*=.044 vs −9.94 [3.86], *P*=.015), MEP (mean difference [SE], −0.88 [3.99], *P*=.828 vs −6.38 [3.99], *P*=.121), %FVC (mean difference [SE], −19.63 [5.13], *P*=.001 vs −21.13 [5.13], *P*<.001) and %PEFR (mean difference [SE], −2.38 [6.76], *P*=.728 vs −11.81 [6.76], *P*=.091). In addition, there was no significant difference between DIS and the Triflow incentive spirometer after the intervention program (*P*>.05; Table 3).

**Table 3.** Comparison in pulmonary function and respiratory muscle strength between a digital incentive spirometer and incentive spirometer among patients who underwent open-heart surgery.

Variable	Group		Mean difference (SE)	*P* value between groups
	DIS^a^ (n=16)	Triflow IS^b^ (n=16)	(DIS-Triflow IS)	
MIP^c^ (cmH_2_O)				
Preoperative, mean (SD)	42.69 (31.06)	46.25 (23.11)	−3.56 (9.68)	.715
Postoperative, mean (SD)	34.56 (24.92)	36.31 (20.33)	−1.75 (8.04)	.829
Mean difference (after-before) (SE)	−8.13 (3.86)	−9.94 (3.86)	N/A^d^	N/A
*P* value within the group	.044	.015	N/A	N/A
MEP^e^ (cmH_2_O)				
Preoperative, mean (SD)	33.69 (17.43)	43.94 (22.45)	−10.25 (7.11)	.159
Postoperative, mean (SD)	32.81 (15.08)	37.56 (15.52)	−4.75 (5.41)	.387
Mean difference (after-before) (SE)	−0.88 (3.99)	−6.38 (3.99)	N/A	N/A
*P* value within the group	.828	.121	N/A	N/A
FVC^f^ (%)				
Preoperative, mean (SD)	68.25 (19.73)	62.50 (16.73)	5.75 (6.47)	.381
Postoperative, mean (SD)	48.63 (20.26)	41.38 (20.67)	7.25 (7.24)	.324
Mean difference (after-before) (SE)	−19.63 (5.13)	−21.13 (5.13)	N/A	N/A
*P* value within the group	.001	<.001	N/A	N/A
PEFR^g^ (%)				
Preoperative, mean (SD)	47.56 (22.05)	44.63 (20.34)	2.94 (7.50)	.698
Postoperative, mean (SD)	45.19 (27.18)	32.81 (21.56)	12.38 (8.67)	.164
Mean difference (after-before) (SE)	−2.38 (6.76)	−11.81 (6.76)	N/A	N/A
*P* value within the group	.728	.091	N/A	N/A

a DIS: digital incentive spirometer.

b IS: incentive spirometer.

c MIP: maximal inspiratory pressure.

d N/A: not applicable.

e MEP: maximal expiratory pressure.

f FVC: forced vital capacity.

g PEFR: peak expiratory flow rate.

## Discussion

### Principal Findings

Herein, we aimed to explore the effects of using the DIS on the respiratory performance of patients who underwent OHS. Based on our findings, the pulmonary function (predicted %FVC and %PEFR) and respiratory muscle strength (MIP and MEP) values did not differ significantly between the DIS and Triflow incentive spirometer groups.

### Pulmonary Function and Respiratory Muscle Strength

Reduced respiratory performance after heart surgery has been well documented [12-14]. The results of the study are in line with those of previous studies on pulmonary function after heart surgery. Narayanan and Hamid [12] reported that the inspiratory capacity dropped to 41% on day 1 post-OHS, gradually increasing to 106% on day 5 post-OHS. In patients who underwent valvular surgery, Alaparthi et al [13] found that pulmonary function (FEV1, FVC, and PEFR) values were reduced when compared with preoperative values and these values improved after day 7 postoperation.

Reportedly, patients who undergo OHS experience functional impairment including functional capacity, physical performance, and psychological health problems [14]. In the present study, pulmonary function (ie, predicted %FVC and %PEFR) and respiratory muscle strength values 5 days postextubation remained lower than the preoperative values. However, the benefit of a DIS gradually decreased when compared with that in the incentive spirometer group. The DIS device features specific attributes that enable the display of numerical values of visual and auditory elements as real-time feedback. Therefore, the use of this DIS device can enhance motivation while performing breathing exercises. Likewise, patients who underwent CABG and used an incentive spirometer experienced a markedly smaller reduction in pulmonary function values than those who performed only deep breathing therapy postoperatively [2,15]. Additionally, a systematic review and meta-analysis of postoperative cardiac surgery (n=1677 participants) revealed a small or no difference in incentive spirometer and standard respiratory care for postpulmonary complications (eg, atelectasis, pneumonia, and mortality) and lung function (eg, FVC, vital capacity, and FEV1), MIP, length of ICU stay or length of hospital stay after cardiac surgery [16]. In other words, the incentive spirometer is not superior to standard respiratory care in terms of clinical outcomes in postoperative cardiac surgery. However, another systematic review and meta-analysis in postoperative care in patients in the incentive spirometer group and without incentive spirometer group reported that patients undergoing incentive spirometryr had significantly shorter reduced risk of postpulmonary complications, and postoperative pneumonia compared with the control group without using IS [17]. Using incentive spirometers may be appropriate for lung re-expansion and oxygen uptake by taking long, slow deep breaths and a breath-hold of 3‐5 seconds resulting in improved collateral ventilation and alveoli [18]. Therefore, a DIS, compared with incentive spirometers, may be an alternative tool for improving respiratory performance and preventing pulmonary complications.

Considering the limitations of this study, we could not compare participants who received the intervention (DIS or Triflow incentive spirometer) and those who did not receive any intervention. The severity of pulmonary function at baseline was not excluded from the study, which may have impacted the results. However, no significant differences were observed between the DIS and Triflow incentive spirometer at baseline. Additionally, the study had a short intervention period because it was conducted at a hospital. Finally, the medical reports and laboratory data were not recorded (eg, comorbidities, duration of operation, ejection fraction, or functional classification), which might affect the baseline characteristic data or initial health status.

### Conclusion

There is a significant decrease in pulmonary function and respiratory muscle strength in the DIS and Triflow incentive spirometer on the 5th day postextubation when compared with preheart surgery values. Additionally, the effect of a DIS on pulmonary function and respiratory muscle strength was similar to a flow-oriented incentive spirometer. Therefore, the innovation of DIS motivates patients to increase their inspiratory effort, thereby encouraging deeper breathing.

## Supplementary material

10.2196/68158 Checklist 1 CONSORT E-Health Checklist

## Abbreviations

CABG: coronary artery bypass graft
DIS: digital incentive spirometer
ERS: European Respiratory Society
FEV1: forced expiratory volume in the 1st second
FVC: forced vital capacity
IS: incentive spirometer
MEP: maximal expiratory pressure
MIP: maximal inspiratory pressure
OHS: open-heart surgery
PEFR: peak expiratory flow rate
